# The effect of an IoT-based intelligent infusion monitoring system on the intravenous infusion safety of hospitalized patients: a retrospective study

**DOI:** 10.3389/fmed.2025.1709955

**Published:** 2025-12-10

**Authors:** Yahui Tong, Xi Pan, Wenjie Sui, Xiaoqing Shi, Lifen Mao, Simin Cheng, Lan Xu

**Affiliations:** The First Affiliated Hospital of Soochow University, Suzhou, China

**Keywords:** intravenous infusion, digital health, patient safety, hospital IoT, infusion monitoring, patient satisfaction

## Abstract

**Background:**

Intravenous infusion is a key part of nursing practice, and its scientific reliability and safety directly affect nursing quality and patient satisfaction. Currently, most hospitals still use traditional gravity infusion, with inherent limitations that burden nursing staff. Therefore, information management is an effective strategy to enhance medication safety.

**Aim:**

This study aimed to explore the effect of intelligent infusion monitoring systems in improving patient infusion safety.

**Methods:**

The study used a retrospective study design. A collaborative development of an intelligent infusion monitoring system using Internet of Things (IoT) technology was conducted. Data from two pilot wards were retrospectively analyzed, including on-time infusion completion rates, infusion-related complications, and patient satisfaction 1 year before and after system implementation. Additionally, infusion abnormalities and nurses’ system satisfaction were recorded during application.

**Results:**

After the application of the intelligent infusion monitoring system, the on-time completion rates of patient infusions significantly improved in both Ward A and Ward B independently, as well as in both wards combined. The occurrence of complications related to infusion significantly reduced in Ward A and in both wards combined. Patient satisfaction also improved after the system application. Most infusion abnormality alerts (74.33%, 85,322 cases) involved blockages or rate abnormalities, with a mean response time of 2.12 min. Nurses’ satisfaction with the system was 75% (33/44).

**Conclusion:**

The application of the intelligent infusion monitoring system effectively improved the on-time completion rates of intravenous infusions and effectively reduced the incidence of complications related to infusion. Through real-time monitoring and alert functions, the system facilitated the timely detection and management of infusion abnormalities, enhancing the quality of infusion management for patients, ensuring infusion safety, and improving patient satisfaction. However, there is still room for improvement in the intelligence and convenience of the system. Therefore, future efforts should focus on further research and investment in intelligent infusion equipment to improve the safety of clinical patient infusions and enhance nursing efficiency.

## 1 Introduction

Intravenous infusion is a method of administering large volumes of sterile fluids, electrolytes, and medications into the body through veins, utilizing the principles of atmospheric and hydrostatic pressures (1). Due to its advantages of timely drug delivery and immediate efficacy, intravenous infusion is widely used in clinical practice, playing a crucial role in patient treatment and care (2). Studies have revealed that the utilization rate of intravenous infusion exceeds 70% in clinical nursing practice in China. This forms an integral part of nursing work and directly impacts the quality of nursing care and patient satisfaction (3, 4). Currently, most hospitals still rely on traditional gravity-based infusion methods, which have inherent weaknesses. Unstable, imprecise infusion rates compromise efficacy, while infusion-related risks (e.g., rate abnormalities, blockages, leakage, air entry, delayed termination) may cause phlebitis, thrombosis, and other complications (5). Additionally, essential infusion parameters, including start time, flow rate, end time, and caregiver information, are manually recorded on paper-based infusion observation cards, posing challenges in post-data analysis and quality control management (6). Challenges arising during the infusion process are identified and addressed primarily through manual patient and family member calls and ward rounds rather than through real-time monitoring of the infusion status (4). With the increasing number of patients and the significant rise in infusion volumes, nursing personnel may experience increased workloads and demands. Consequently, improving nursing efficiency and enhancing the safety and reliability of intravenous infusion therapy are imperative clinical concerns.

With the rapid development of information technology in the healthcare sector, leveraging information systems for automatic data collection and analysis, guiding clinical practice, and ensuring patient safety has become the current trend. Information management is considered an effective measure to promote medication safety (4, 7). The “Action Plan for Further Improving Nursing Services (2023–2025)” issued by the National Health Commission (8) emphasizes that healthcare institutions should accelerate the development of nursing informatization through the construction of smart hospitals, smart wards, and full integration of new-generation information technologies such as artificial intelligence, 5G, and the Internet of Things (IoT). This will improve and optimize nursing service processes, enhance nursing efficiency, and alleviate the workload of frontline clinical nurses. Therefore, this paper highlights the need and importance of nursing informatization.

Currently, research on intravenous infusion information systems primarily depends on personal digital assistants (PDAs) to facilitate partial monitoring of the infusion process, such as medication administration and verification. This has reduced manual recording time, effectively improved work efficiency, reduced the occurrence rate of medication errors related to infusion, and enhanced nursing quality (9, 10). However, this approach only captures fragmented information and lacks real-time and comprehensive monitoring of infusion data. Therefore, developing an IoT-based intravenous infusion monitoring system to address these clinical gaps and meet national requirements for hospital information system (HIS) intelligent construction is imperative.

For the above reasons, we collaborated with Chongqing Sinocloud Technologies Ltd., to develop an intelligent system that utilizes IoT and information technology to assist intravenous infusion. This system was tested in orthopedic and neurology departments in September 2022, and its impact on patient infusion safety and nursing efficiency was investigated. The research findings are presented below.

## 2 Methods

### 2.1 Study design

The study used a retrospective study design. Retrospective studies have inherent causal inference limitations, as potential confounding factors cannot be fully excluded. We controlled for seasonal confounders by matching pre-intervention and post-intervention seasonal distributions and adjusted for residual confounders in statistical analyses.

### 2.2 Research setting and objects

This study selected two pilot wards in a tertiary hospital in Jiangsu Province, China, as the research subjects: the neurology department (Ward A) and the orthopedic department (Ward B). They had 53 and 46 open beds, respectively, resulting in a total of 99 beds. Before and after the system application, the number of nursing staff in Ward A was 25 both periods. Two nurses underwent rotation; the nurses transferred out and in had the same staff levels (N1 and N0) and the same professional titles (staff nurse and senior staff nurse). For Ward B, the number of nurses was 18 before the system application and 19 after, with one additional staff nurse at the N0 level added post-application. Overall, the changes in nursing manpower in both wards were minimal before and after the system application. Inclusion criteria: Inpatients who received intravenous infusion in the two pilot wards during the study period; complete medical records and infusion data. Exclusion criteria: Patients transferred midway, those who refused to participate in satisfaction surveys, and cases with missing key infusion data.

### 2.3 Design of intelligent infusion monitoring system

The system is an intelligent system developed by Chongqing Xinan Bijie iot Technology Co., LTD, which utilizes IoT and information technology to assist with intravenous infusion. The system can provide statistical analysis of patient infusion data, real-time monitoring of patient infusion status, and parsing and storage of relevant data in the database to generate new information. The intelligent infusion monitoring system mainly comprises four parts: mobile infusion equipment, infusion client, infusion server, and HIS.

**Figure 1:**
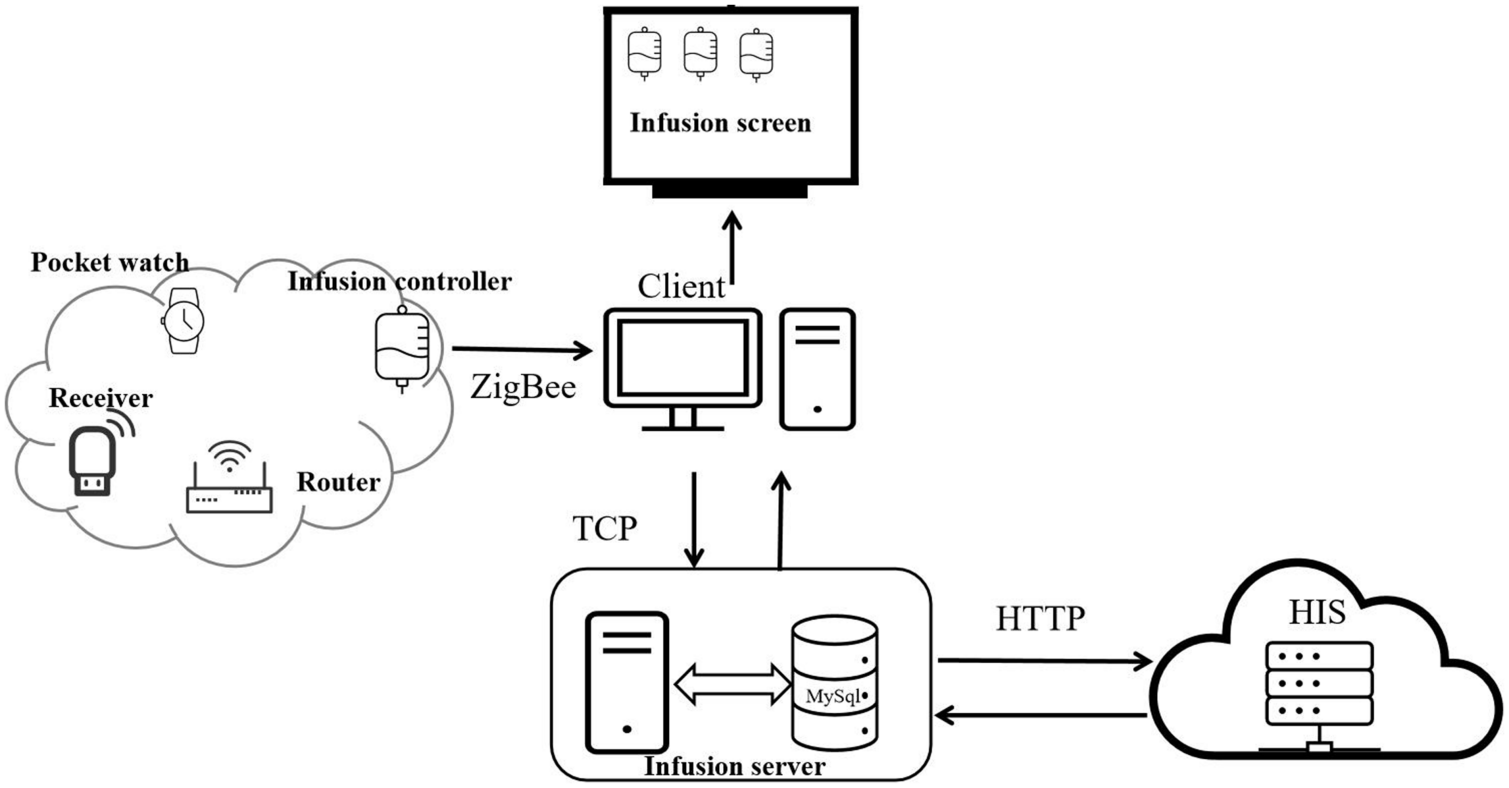
The operation process of the system.

Operation process of the system (Figure 1): The system collects and monitors patient infusion data (flow rate, status, and total infused volume) using mobile infusion equipment (comprising flow rate infusion controllers, routers, receivers, and wearable hanging watches). Among them, the mobile infusion equipment adopts advanced electromagnetic anti-interference and Digital Signal Processor (DSP) droplet detection technology. The DSP processor processes signals collected by the droplet sensor, enabling high-precision and highly stable droplet detection and control, which provides core technical support for accurate collection of infusion data.

The mobile infusion equipment stores and transmits the collected infusion data in real time through a Zigbee wireless network, sending information such as current infusion status, total infused volume, ward number, and bed number to the infusion client. Subsequently, the infusion client interacts with the infusion server via the Transmission Control Protocol (TCP) — during this process, data is encrypted for transmission. This interaction not only enables the upload of infusion data to the infusion server but also completes the acquisition of patient information and medical order information from the infusion server.

**Figure 2:**
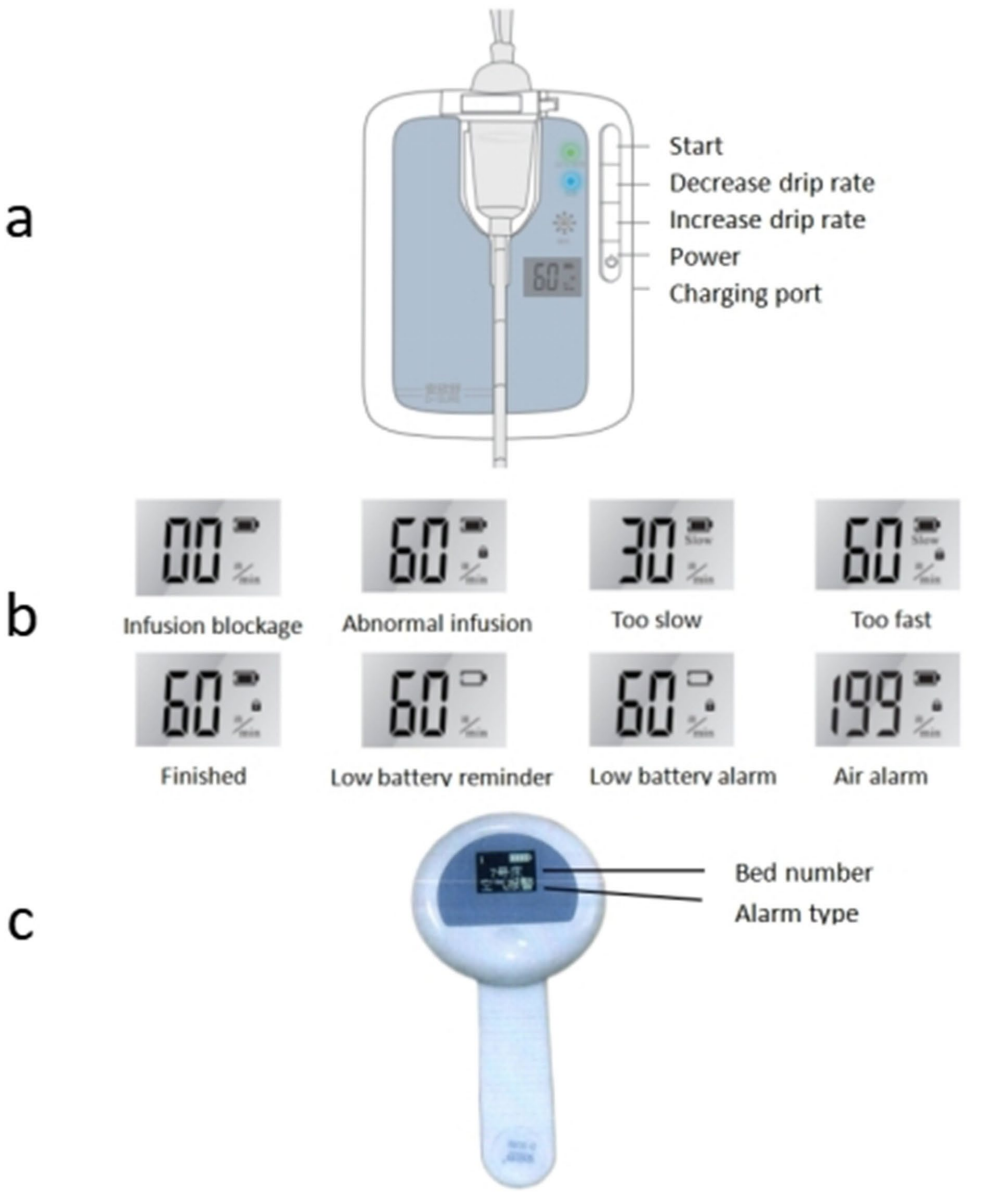
Mobile infusion equipment alarm status [**(a)** flow rate infusion controllers; **(b)** abnormal infusion alarm; **(c)** wearable hanging watches].

After obtaining the infusion data from the mobile infusion equipment and patient information from the infusion server, the infusion client synchronizes the relevant data to the large infusion display screen. It displays real-time information about each patient’s bed, medical orders, and current infusion status, and then issues corresponding prompts and alarms based on the infusion status. Alarm thresholds are set by drug type and volume: ±15% for routine drugs, ±10% for high-risk drugs, and ±5% for volumes ≤50 mL. Exceeding thresholds triggers automatic alarms, with flow rate controllers and nurses’ wearable hanging watches providing timely notifications (Figure 2).

Throughout the process, the infusion server achieves bidirectional data interaction with the hospital’s Hospital Information System (HIS) via the Hypertext Transfer Protocol (HTTP): the HIS system can transmit ward, patient, and medical order information to the infusion server, while the infusion server can feed back alarm data to the HIS system. All relevant information in the infusion server is stored in a MySQL database. Users and administrators can query detailed historical infusion records of each ward through the software backend, including key data such as infusion time, infused volume, number of alarms, and response time.

**Figure 3:**
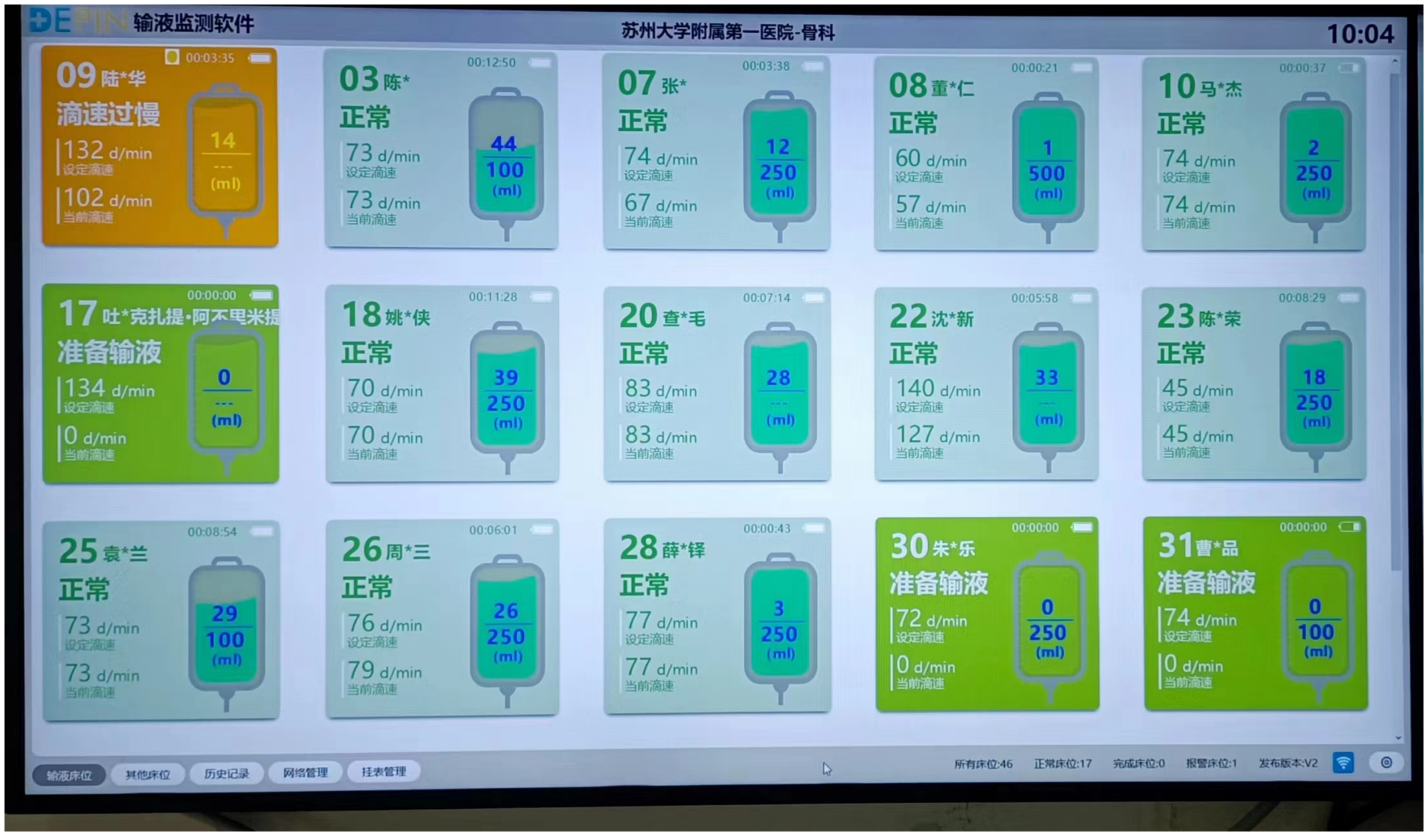
Infusion screen.

Different infusion statuses on the large infusion screen are visually indicated and accompanied by audio-visual alarms: (1) “Preparation for infusion” displays in green; (2) “Normal infusion” display in light green; (3) “Infusion completed” displays in light yellow, with yellow indicator light flashing and audio alarm; (4) “Infusion about to complete” displays in light yellow, with audio prompt; (5) “Infusion blockage” displays in yellow, with yellow indicator light flashing and audio alarm; (6) “Drip rate too slow” displays in yellow, with yellow indicator light flashing and audio alarm; (7) “Drip rate too fast” displays in orange-yellow, with yellow indicator light flashing and audio alarm; (8) “Air alarm” displays in orange-yellow, with yellow indicator light flashing and audio alarm; (9) “Infusion anomaly” displays in orange-yellow, with yellow indicator light flashing and audio alarm; (10) “Low battery level” displays in orange-yellow, with yellow indicator light flashing and audio alarm (Figure 3).

### 2.4 Application security

During system usage, in the event of an abnormal shutdown of the system, the flow rate infusion controller monitors and stores infusion data in real time. Once the system is restarted, it synchronizes the current data to the database and displays it in real time on the system interface. The database has backup mechanisms to ensure data integrity. Bed number and drip rate configuration take ≤20 s from “Start” button press to display on the nursing screen. In the event of infusion completion or infusion anomaly, the flow rate infusion controller can automatically control and lock the infusion tubing in a timely manner to prevent infusion accidents.

### 2.5 Network security

The system can only access authorized wards added by authorized personnel in the server’s backend, and unauthorized personnel cannot add wards. The data interaction between the infusion server and infusion client uses the TCP, and these data are transmitted after encryption to ensure the reliability. Authorized users can access the corresponding functions based on their permissions upon successfully logging into the software. Logs are provided to record unusual access information and user behavior.

### 2.6 Interventions

In September 2022, the intelligent infusion monitoring system was installed and tested in the neurology and orthopedic departments, with 53 and 46 mobile devices in each ward, resulting in a total of 99 devices.

System operation process: (1) Device installation: Before starting the infusion, the nursing staff opens the detection clamp on the mobile infusion equipment, clamps the upper part of the burette, and ensures that it contains either half of the liquid with the liquid level below the lower side of the clamp. One hand holds the bottom of the infusion tube while the other straightens it slightly into the groove, ensuring that the infusion tube is properly inserted into the groove. (2) Device operation: Briefly press the “Power” button to start the device, after which the last set bed number is displayed. Change the bed number using the “Decrease flow rate/Increase flow rate” buttons and press the “Start” button upon completion. After setting the bed number, the device automatically switches to the set flow rate interface, and the flow rate is set using the “Decrease drip rate/Increase drip rate” buttons. After setting the flow rate, briefly press the “Start” button. The device will automatically adjust the infusion rate, with liquid drops and green light flashing simultaneously. Once the device starts operating normally, the one-time infusion drip adjuster should be opened completely. When the nursing staff completes the infusion operation, they can use the nursing whiteboard at the nursing station or the wearable hanging watch to learn about the patient’s infusion status at any time. When the nursing whiteboard or wearable hanging watch alerts that the patient’s infusion is blocked, too fast, too slow, or completed, the nursing staff can immediately go to the patient’s bedside for further action. (3) Adjusting the drip rate: During the infusion, if there is a need to change the drip rate, briefly press the “Start” button to enter the pause mode, modify the drip rate, and then briefly press the “Start” button. The device will automatically adjust to the new drip rate.

### 2.7 Resource allocation and operation management

To ensure system stability, the following standardized management was implemented. (1) Nurse training: 44 nurses (25 in Ward A, 19 in Ward B) received 396 h of stratified training (August 2022): basic operation, alarm handling, maintenance, with 100% assessment pass rate. (2) Device maintenance: For 99 devices (53 in Ward A, 46 in Ward B), daily inspection (16.5 h/day, 495 h/month) and monthly calibration (99 h/month, 1,188 h/year) were conducted.

### 2.8 Instruments

#### 2.8.1 Comparison of total infusion amount before and after the use of intelligent infusion monitoring system in wards

A Comparison of the quarterly average number of inpatients and quarterly average intravenous infusion volume in the wards before and after the system application was made. Relevant data were exported from the hospital’s HIS system and the Medi-Link system.

#### 2.8.2 Comparison of timely infusion completion rate before and after the use of intelligent infusion monitoring system in wards

A comparison of the timely infusion completion rate in the wards before and after the system application was done. Timely Infusion completion rate was defined as the proportion of infusions with actual duration within the expected range (calculation: on-time infusions/total infusions × 100%). Expected duration was determined by medical orders, drug characteristics, and “Fundamentals of Nursing” (11):. For routine drugs (without special medical orders), the interval value was calculated as “volume × drip rate coefficient (15 or 20 drops/mL) ÷ standard drip rate (40–60 or 53–80 drops/min).” For special drugs/patients (with clear medical orders), the fixed value was determined by the medical order time or “volume × drip rate coefficient ÷ medical order drip rate.” The allowable deviation range was set at ±15%, reduced to ±10% for high-risk drugs, and to ±5% for ≤50 mL of liquid. The start and end times of each infusion were recorded through PDA scanning (the Medi-Link system automatically calculated the actual infusion duration). If the actual duration fell within the range of “expected duration × (1 ± deviation ratio),” it was considered completed on time; if exceeded (including premature completion, as it may increase the risk of adverse reactions), it was considered delayed.

#### 2.8.3 Comparison of intravenous infusion complications before and after the use of intelligent infusion monitoring system in wards

A comparison of the occurrence of infusion-related complications in the wards before and after the system application was conducted. Infusion-related complications included phlebitis, infusion extravasation (divided into drug infiltration and drug extravasation based on whether the drug is corrosive), catheter-related bloodstream infection (CRBSI), catheter-related thrombosis (CRT), and others.

Specific diagnostic criteria for each complication were adopted: Phlebitis was evaluated using a progressive scoring system (0–4 points) as specified in the INS Guidelines, with a score of ≥1 defined as phlebitis (12, 13). Drug infiltration refers to the entry of non-corrosive solutions into peripheral tissues outside the venous lumen during intravenous therapy, while drug extravasation involves corrosive solutions entering such tissues (14). CRBSI was diagnosed when patients with an intravascular catheter (or within 48 h of catheter removal) developed bacteremia/fungemia with infectious manifestations (e.g., fever, chills, hypotension) without other definite infection sources, and microbiological tests confirmed positive peripheral blood cultures or consistent pathogenic bacteria in catheter segment and peripheral blood cultures (14). CRT included four categories, and the complication involved in this study was mainly thrombotic catheter dysfunction (impaired or complete transcatheter infusion occlusion caused by fibrin sheath, intraluminal thrombosis, or catheter tip thrombosis) (15).

When suspected infusion-related complications occurred, bedside nurses initially reported them to the department liaison nurse for preliminary judgment. Confirmed cases were reported through the infusion-related complications reporting system (HaiTai), with submitted content including patient basic information, catheter type and indwelling time, complication type, occurrence time, site, and relevant photos. The leader of the hospital’s Intravenous Therapy Nursing Team then conducted a final confirmation. The backend of the HaiTai system could uniformly export the reported data for statistical analysis. During the study period, the above complication identification and reporting workflow remained unchanged.

#### 2.8.4 Comparison of patient satisfaction before and after the use of intelligent infusion monitoring system in wards

Evaluation of patient satisfaction in the wards before and after the system application was conducted using a self-made patient satisfaction questionnaire that has been long-term used for patient satisfaction surveys in our hospital. The questionnaire consists of 20 items, mainly covering patients’ satisfaction with various nursing services, including medication administration, diet, ward environment, disease education, service attitude, fluid replacement, rehabilitation guidance, discharge guidance, and nursing operation skills. It adopts a 5-point Likert rating scale, with scores ranging from 1 to 5, representing “very dissatisfied,” “dissatisfied,” “neutral,” “satisfied,” and “very satisfied,” respectively. The total score is 20–100 points, with a higher score indicating higher satisfaction. The Cronbach’s *α* coefficient of the questionnaire is 0.924, indicating good reliability. Before discharge, bedside nurses guide patients to complete the anonymous satisfaction questionnaire through the “BoXi Nursing” WeChat official account, and the backend system uniformly exports the patient satisfaction scores.

#### 2.8.5 Number of abnormal infusion alerts and average response time

Recording of abnormal infusion alert events detected by the system and nurse response time. When nurses perform intravenous infusion procedures and enter the required drip rate into the drip rate controller, the system will generate an alert if the detected drip rate exceeds the set range by 15% during the infusion process and will record the relevant events. When nurses detect the alert, they will go to the bedside to resolve the alert and take appropriate action. The system will automatically record the time difference from when the alert was issued to when it was resolved by the nurse as the nurse’s response time.

#### 2.8.6 Nurse satisfaction after the use of intelligent infusion monitoring system in wards

Evaluation of nurse satisfaction in the wards after the system application was conducted using a self-developed Nurse Satisfaction Questionnaire. The questionnaire consists of 10 items, mainly covering nurses’ evaluations of the infusion monitoring system, including ease of use, clear display of key information, alarm accuracy, convenience of data query, work efficiency improvement, and operational stability. Each item adopts a 5-point Likert scale with options: “very dissatisfied,” “dissatisfied,” “neutral,” “satisfied,” and “very satisfied,” resulting in a total score ranging from 10 to 50 points—a score of ≥40 indicates satisfaction, while a score of <40 indicates dissatisfaction. The Cronbach’s *α* coefficient of the questionnaire is 0.881, demonstrating good reliability. Nurses completed the satisfaction questionnaire anonymously online by scanning a QR code via WeChat, and the satisfaction results could be uniformly exported from the backend. The number of satisfied respondents was calculated by categorizing “satisfied” and “very satisfied” as positive responses. Nurse satisfaction was computed using the formula: Nurse satisfaction = (Number of satisfied respondents/Total number of nurses participating in the evaluation) × 100%.

### 2.9 Data collection

**Figure 4:**
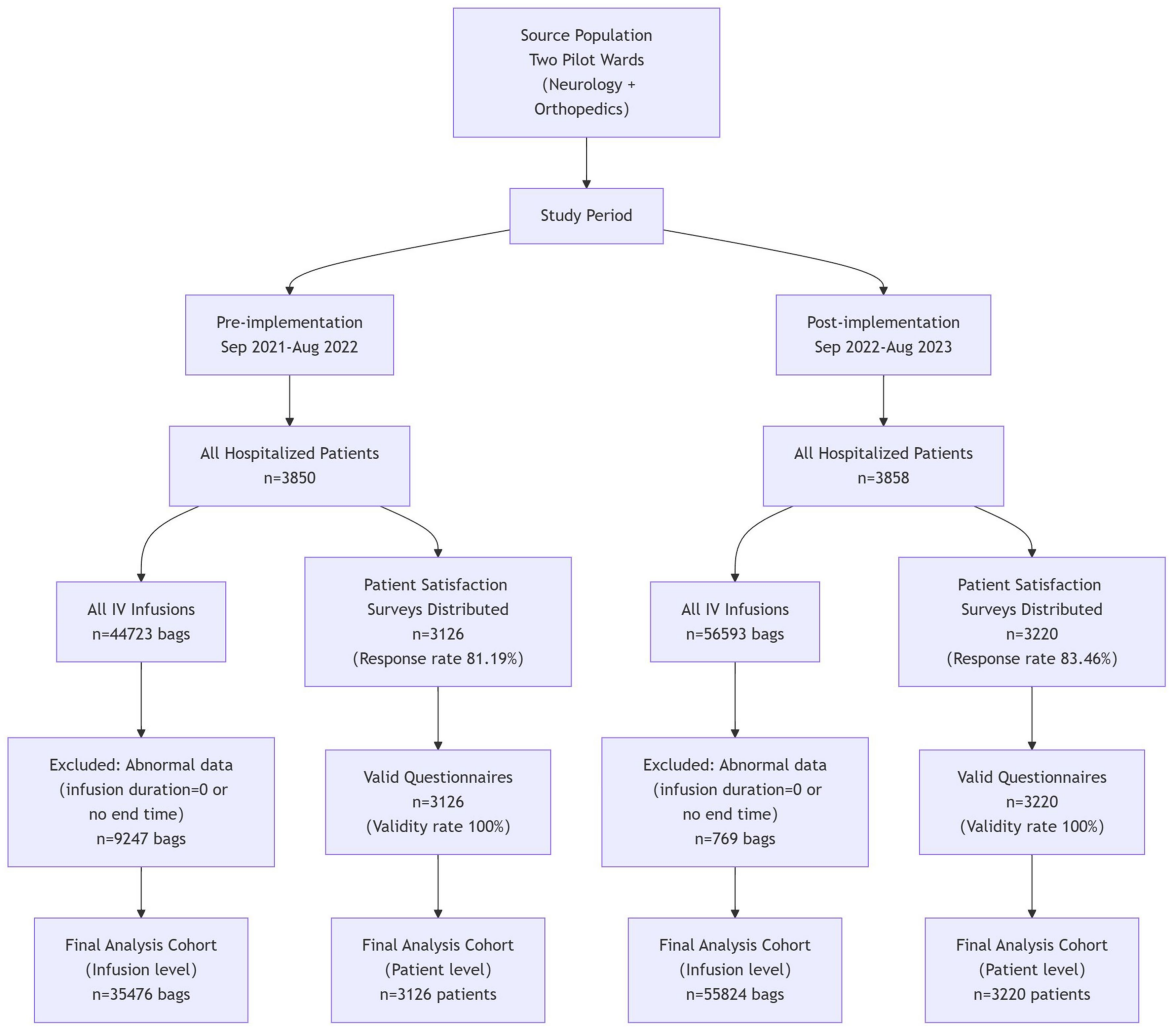
The study flow diagram.

The researchers collected data on the completion of on-schedule intravenous infusion, occurrence of complications related to intravenous infusion, patient satisfaction, the average number of inpatients, and the average total volume of intravenous infusion from the Medi-Health System, HaiTai System, “BoXi Nursing” official account, and HIS System. Data were collected over two 1-year cycles: September 2021–August 2022 (pre-system application) and September 2022–August 2023 (post-system application), with consistent seasonal distribution across the two phases to control for seasonal confounders. The number of inpatients in the two wards before and after the application of the systems was 3,850 and 3,858, respectively. A total of 3,126 and 3,220 patient satisfaction questionnaires were collected before and after application, with response rates of 81.19 and 83.46%, respectively, and 100% of the questionnaires were valid. The data on abnormal infusion alert frequency and average response time were exported from the infusion monitoring system collected from September 1, 2022, to August 31, 2023. The survey on nurse satisfaction was conducted by distributing questionnaire star QR codes to various ward nurse work groups. Data collection was done from May 15, 2024, to May 22, 2024, with 44 valid responses and a 100% response rate (Figure 4).

### 2.10 Statistical analysis

For statistical analysis, Statistical Package for Social Sciences software (version 26.0) was used. Continuous data are expressed as mean ± standard deviation and analyzed using independent sample *t*-tests. Categorical data are expressed as frequency, percentage, or rate analyzed using the Chi-square test. A *p* < 0.05 was considered statistically significant.

In this study, an interrupted time series (ITS) model was employed, with the “continuous number of months since the baseline month of the study initiation” as the time variable (Time) and “whether the infusion monitoring system intervention was implemented” as the intervention indicator (Intervention, coded as 0 for the pre-intervention period and 1 for the post-intervention period). The post-intervention trend term was defined as the interaction term between the time variable and the intervention indicator (Time×Intervention), serving to characterize the intervention’s impact on the long-term trend of the outcome indicator. Seasonal terms were excluded from the model, and the Newey-West method was applied to address autocorrelation issues. The final form of the model was expressed as: Y = β_0_ + β_1_ × Time+β_2_ × Intervention+β_3_ × (Time × Intervention) + *ε*. Here, ε denotes the error term; β_0_ represents the model intercept, corresponding to the level of the outcome indicator in the baseline month of the study; *β*_1_ stands for the pre-intervention trend, indicating the monthly change magnitude of the outcome indicator before intervention implementation; *β*_2_ refers to the level change, capturing the immediate change magnitude of the outcome indicator at the intervention initiation point; β_3_ denotes the post-intervention trend change, representing the difference in monthly change magnitude between the post-intervention and pre-intervention periods, with β_1_ + β_3_ being the post-intervention slope. All coefficients are expressed in the unit of “percentage points per month.”

### 2.11 Ethical considerations

This study has been approved by the hospital Ethics Committee of the First Affiliated Hospital of Soochow University (ethics number: 2024075). The need for informed consent was waived by the ethics committee due to the retrospective nature of the study. All patient data were anonymized during collection and analysis to protect patient privacy. Data storage and transmission complied with national data security regulations, and only authorized researchers had access to the original data. The infusion controller involved in this study is an approved medical device with the registration number “Yu Xie Zhu Zhun 20172540087.” This product was approved and issued a certificate by the Chongqing Municipal Drug Administration.

## 3 Results

### 3.1 Comparison of quarterly inpatient numbers and quarterly intravenous infusion volumes before and after system application

Independent sample *t*-test was used for comparison between groups. The comparison of inpatient numbers and intravenous infusion volumes before and after system application was conducted on a quarterly basis. The results revealed no statistically significant difference (*p* > 0.05) in the quarterly inpatient numbers and quarterly intravenous infusion volumes between the year before system application and the year after system application, indicating comparability, as presented in Table 1.

**Table 1:** Comparison of quarterly inpatient numbers and quarterly intravenous infusion volumes before and after system application.

Items	Objects	1 year before application	1 year after application	*t*	*p*
Quarterly inpatient numbers	Ward A	470.50 ± 16.34	507.25 ± 25.40	−2.434	0.051
	Ward B	492.00 ± 28.13	457.25 ± 79.15	0.827	0.440
	Both wards	481.25 ± 24.20	482.25 ± 60.63	−0.043	0.966
Quarterly intravenous infusion volumes	Ward A	17986.67 ± 4779.54	24,265 ± 981.73	−2.229	0.090
	Ward B	9692.50 ± 1264.01	11344.75 ± 1262.55	−1.85	0.114
	Both wards	13247.14 ± 5297.99	16,882 ± 6986.66	−1.097	0.294

### 3.2 Comparison of timely completion rate of intravenous infusion

Chi-square test was used for comparison of rates between groups; sensitivity analysis was conducted for missing data; interrupted time series (ITS) model with Newey-West correction was used to analyze trend changes; regression analysis was used to analyze the relationship between the two variables of timely completion rate of intravenous infusion and intravenous infusion-related complications, and subgroup analysis was used to explore the heterogeneity of the variables. Due to the large volume and diverse types of medications involved in intravenous infusion in this study, to facilitate the statistical data and requirements of infusion time and drip rate, we selected commonly used medications in each ward. These medications accounted for over 50% of the total infusion volume in the ward each year. There were a total of 16 types of medications, with 11 for Ward A and 5 for Ward B. The screened infusion volumes before application were 44,723 bags (Ward A: 24,933 bags; Ward B: 19,790 bags), and after application, were 56,593 bags (Ward A: 33,001 bags; Ward B: 23,592 bags). After excluding some abnormal data (infusion start time and end time difference of 0; no end time), the final data obtained were as follows: 35,476 bags before application and 55,824 bags after application, with valid data rates of 79.32% (35,476/44,723) and 98.64% (55,824/56,593), respectively. Through comparison, the results revealed significant differences in the timely completion rate of intravenous infusion in Ward A, Ward B, and both wards before and after the system application (*p* < 0.001) (Table 2).

**Table 2:** Comparison of timely completion rate of intravenous infusion (%).

Situation	Objects	Before application (baseline value) [Timely completion rate (number of cases)]	After application (observation value) [Timely completion rate (number of cases)]	Chi-square	*p*	Absolute risk difference (compared to the baseline)	RR (95% CI)
Valid data (actual observation)	Ward A	28.39 (4,551/16,032)	38.58 (12,636/32,754)	489.95	<0.001	10.19	1.359 (1.321–1.399)
	Ward B	41.52 (8,074/19,444)	59.52 (13,731/23,070)	1,400	<0.001	18.00	1.433 (1.404–1.462)
	Both wards	35.59 (12,625/35,476)	47.23 (26,367/55,824)	1,200	<0.001	11.64	1.327 (1.305–1.349)
Best-case scenario (missing data is categorized as ‘Completed on Time’)	Ward A	54.00 (13,452/24,933)	39.04 (12,883/33,001)	1274.204	<0.001	−14.96	0.724* (0.711–0.736)
	Ward B	42.55 (8,420/19,790)	60.41 (14,253/23,592)	1377.192	<0.001	17.86	1.420 (1.393–1.447)
	Both wards	48.91 (21,872/44,723)	47.95 (27,136/56,593)	9.144	0.002	−0.96	0.980 (0.968–0.993)
Worst-case scenario (missing data is categorized as ‘Not Completed on Time’)	Ward A	18.25 (4,551/24,933)	38.29 (12,636/33,001)	2732.735	<0.001	20.04	2.096 (2.037–2.160)
	Ward B	40.80 (8,074/19,790)	58.20 (13,731/23,592)	1303.913	<0.001	17.40	1.427 (1.399–1.456)
	Both wards	28.23 (12,625/44,723)	46.59 (26,367/56,593)	3557.502	<0.001	18.36	1.650 (1.623–1.678)

Outcome coding: Completed on time = 1, Not completed on time = 0; Reference group: Control group.*In Ward A, the timely completion rate decreased after application under the best-case scenario. However, the volume of missing data before the intervention was relatively large (with an original effective data rate of 79.32%). Assuming that all missing data are categorized as “completed on time” would overestimate the baseline value (54.00%), which is inconsistent with the clinical reality where “the probability of incomplete tasks is higher.” This result is mainly affected by data assumptions, rather than a reversal of the system’s effect.

To verify the robustness of the results, a sensitivity analysis was conducted for missing data. Two scenarios were defined: the best-case scenario, where all missing data were assumed to be “completed on time”; and the worst-case scenario, where all missing data were assumed to be “not completed on time.” The results are presented in Table 2. (1) Best-Case Scenario: Using pre-application data as the baseline, in Ward B, the post-application on-time completion rate (14,253/23,592, 60.41%) remained significantly higher than the baseline (8,420/19,790, 42.55%, *p* < 0.001), maintaining an upward trend. In Ward A, however, the post-application rate (12,883/33,001, 39.04%) was significantly lower than the baseline (13,452/24,933, 54.00%, *p* < 0.001). The overall rate (27,136/56,593, 47.95%) was slightly lower than the baseline (21,872/44,723, 48.91%, *p* = 0.002). This discrepancy is primarily attributed to the large amount of missing data in Ward A before the intervention. Assuming all missing data were “completed on time” led to an overestimation of the baseline value (see Note), which deviates significantly from actual clinical practice. A comprehensive assessment should therefore be made by integrating the results of valid data. (2) Worst-Case Scenario: For all analyzed subjects (Ward A, Ward B, and the overall cohort), the post-application on-time completion rates were significantly higher than the pre-application baselines: Ward A: (12,636/33,001) 38.29% vs. (4,551/24,933) 18.25%; Ward B: (13,731/23,592) 58.20% vs. (8,074/19,790) 40.80%; Overall: (26,367/56,593) 46.59% vs. (12,625/44,723) 28.23% (all *p* < 0.001). This is consistent with the upward trend observed in valid data, indicating that even under the most unfavorable data assumptions, the effect of the intelligent infusion monitoring system in improving the on-time completion rate remains robust.

**Figure 5:**
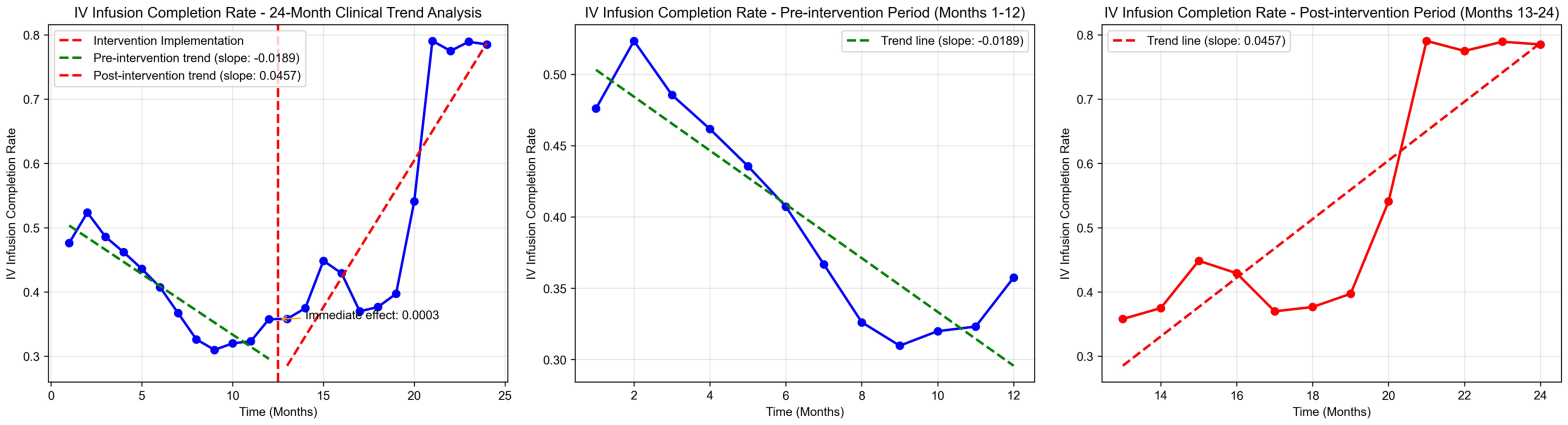
IV infusion completion rate time series analysis.

**Figure 6:**
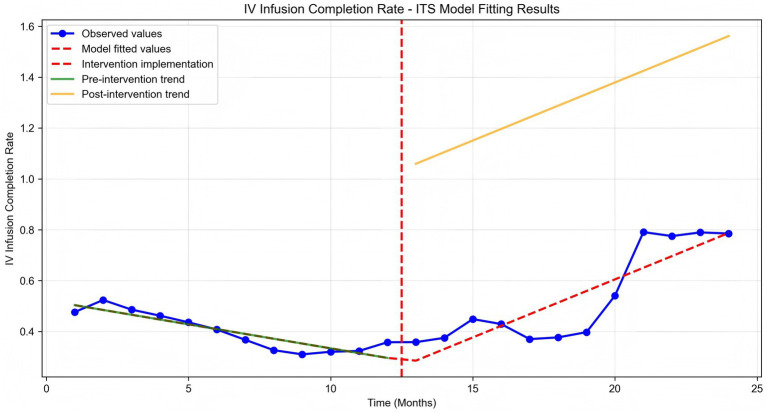
IV infusion completion rate ITS model fitting.

Time series plots were constructed for the timely completion rate of intravenous infusion over 24 months (Figure 5). Results of the interrupted time series model fitted with Newey-West correction for autocorrelation (Table 3; Figure 6) revealed the following: Before the intervention, the monthly timely completion rate of intravenous infusion in the two wards significantly decreased by 1.89 percentage points (β1 = −1.89, SE = 0.30, *p* < 0.001). At the intervention node, there was no significant level jump in the on-time completion rate of the two wards (β2 = −5.63, SE = 5.00, *p* = 0.259). After the intervention implementation, however, the change trend of the timely completion rate of intravenous infusion was significantly improved compared with that before the intervention, with the average monthly change amplitude increasing by 6.45 percentage points relative to the pre-intervention period (β3 = 6.45, SE = 0.90, *p* < 0.001). Specifically, the monthly timely completion rate showed a significant upward trend after the intervention (β₁ + β₃ = 4.56 percentage points/month, *p* < 0.001).

**Table 3:** Coefficients of interrupted time series (ITS) model for on-time completion rate of intravenous infusion and incidence rate of infusion complications in two pilot wards from September 2021 to August 2023.

Model coefficients	Coefficient value	95% CI	Z	*p*
IV infusion completion rate				
β0	52.21	49.00 ~ 55.40	31.737	<0.001
β1	−1.89	−2.40 ~ −1.30	−6.881	<0.001
β2	−5.63	−15.40 ~ 4.20	−1.128	0.259
β3	6.45	4.80 ~ 8.10	7.512	<0.001
IV infusion complication rate				
β0	0.50	0.30 ~ 0.60	6.514	<0.001
β1	0.00	0.00 ~ 0.00	−0.426	0.670
β2	−0.10	−0.30 ~ 0.10	−0.999	0.318
β3	−0.02	−0.10 ~ 0.00	−1.210	0.226

Further subgroup analysis was conducted to explore the heterogeneity of the timely completion rate across wards and drug types. Subgroup by ward: Baseline regression showed a significant negative correlation between on-time infusion completion and complication rate (coefficient = −0.003, *p* < 0.01) (Table 4). However, subgroup analysis by ward revealed distinct results: Ward A showed a significant negative correlation (coefficient = −0.013, *p* < 0.01), while Ward B presented a significant positive correlation (coefficient = 0.006, *p* < 0.05), highlighting the critical role of ward-specific factors (Table 5). Subgroup by drug type: Among 16 types of commonly used drugs, Drug I, L, O were excluded from regression due to no variation in the dependent variable, making it impossible to estimate the association with on-time completion. Among the remaining 13 drugs, only Drug D showed a significant negative correlation between on-time completion and complication rate (coefficient = −0.020, *p* < 0.05). The other 12 drugs showed no significant correlation, indicating that drug type modulates the relationship between completion status and complications (Table 6).

**Table 4:** Baseline regression analysis.

Variables	(1)
	Complication_rate
Completion	−0.003*** (−3.06)
log_pop	−0.832*** (−60.83)
Constant	4.642*** (66.73)
Observations	35,476
R-squared	0.951
Firm FE	Yes
Year FE	Yes
Controls	Yes

*T*-statistics in parentheses. ***p < 0.01.

**Table 5:** Regression analysis by ward.

Variables	(1)	(2)
	Complication_rate	Complication_rate
Completion	−0.013*** (−2.67)	0.006** (2.23)
log_pop	1.169*** (22.26)	−1.342*** (−56.65)
Constant	−5.096*** (−19.11)	6.890*** (57.14)
Observations	16,032	19,444
R-squared	0.031	0.142
Ward month drug FE	Yes	Yes
Controls	Yes	Yes

*T*-statistics in parentheses. ***p < 0.01, **p < 0.05.

**Table 6:** Regression analysis by drug stratification.

Variables	A	B	C	D	M	N	P	E	F	G	H	J	K
	Drug	Drug	Drug	Drug	Drug	Drug	Drug	Drug	Drug	Drug	Drug	Drug	Drug
Completion	0.002 (0.18)	0.016 (0.55)	0.024 (1.39)	−0.020** (−2.03)	0.004 (1.25)	0.005 (0.84)	−0.001 (−0.15)	0.030 (1.23)	0.022 (1.29)	0.021 (0.70)	−0.016 (−0.82)	0.004 (0.22)	0.016 (0.82)
Log_pop	1.506*** (13.76)	−12.114*** (−26.29)	−9.202*** (−34.50)	1.163*** (9.88)	−1.449*** (−43.16)	−1.049*** (−21.59)	−1.332*** (−29.71)	1.635*** (9.23)	0.835*** (4.73)	2.965*** (11.49)	0.125 (0.57)	1.841*** (7.49)	1.161*** (10.54)
Constant	−6.820*** (−12.28)	62.829*** (26.67)	47.965*** (35.24)	−5.089*** (−8.53)	7.445*** (43.47)	5.386*** (21.74)	6.842*** (30.05)	−7.448*** (−8.28)	−3.412*** (−3.80)	−14.172*** (−10.85)	0.150 (0.13)	−8.488*** (−6.81)	−5.066*** (−9.06)
Observations	3,444	279	952	2,723	9,160	3,461	6,823	1,363	1,518	648	772	825	3,442
R-squared	0.052	0.715	0.558	0.036	0.169	0.119	0.115	0.059	0.015	0.170	0.001	0.064	0.031
Ward month drug FE	Yes	Yes	Yes	Yes	Yes	Yes	Yes	Yes	Yes	Yes	Yes	Yes	Yes
Controls	Yes	Yes	Yes	Yes	Yes	Yes	Yes	Yes	Yes	Yes	Yes	Yes	Yes

### 3.3 Comparison of intravenous infusion-related complications

Chi-square test was used for comparison of complication rates between groups; interrupted time series (ITS) model with Newey-West correction was used to analyze trend changes; two-way fixed effect panel regression model was used to analyze the association between drug proportion and complication incidence, and subgroup analysis was used to explore the heterogeneity of drug proportion and infusion complication association in different wards. Comparison of the occurrence of intravenous infusion-related complications before and after the system application in the units showed a statistically significant difference in the total number of occurrences in the Ward A and both wards (*p* < 0.05) (Table 7).

**Table 7:** Comparison of intravenous infusion-related complications.

Objects	Application stage	Intravenous Infusion-Related Complications (*n*)	No Intravenous Infusion-Related Complications (*n*)	Hospitalized patients (*n*)	Chi-square	*p*	RR (95% CI)
Ward A	Before application	17	1,865	1,882	6.165	0.013	0.994 (0.989–0.999)
	After application	6	2,023	2,029			
Ward B	Before application	1	1,967	1,968	0.93	0.335	0.999 (0.998–1.000)
	After application	0	1,829	1,829			
Both wards	Before application	18	3,832	3,850	6.044	0.014	0.997 (0.994–0.999)
	After application	6	3,852	3,858			

**Figure 7:**
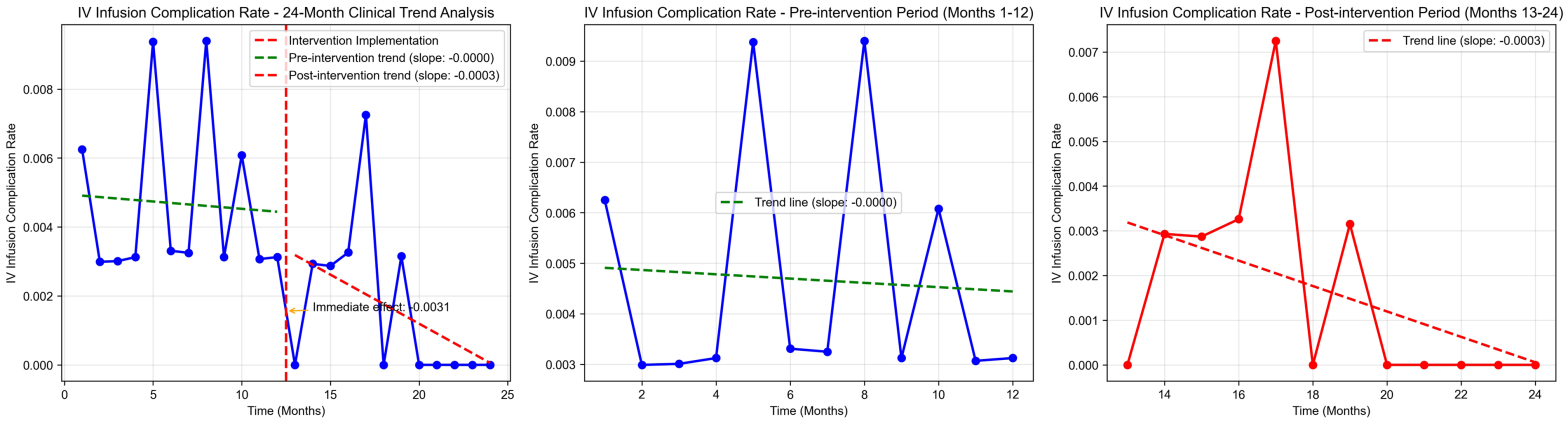
IV infusion complication rate time series analysis.

**Figure 8:**
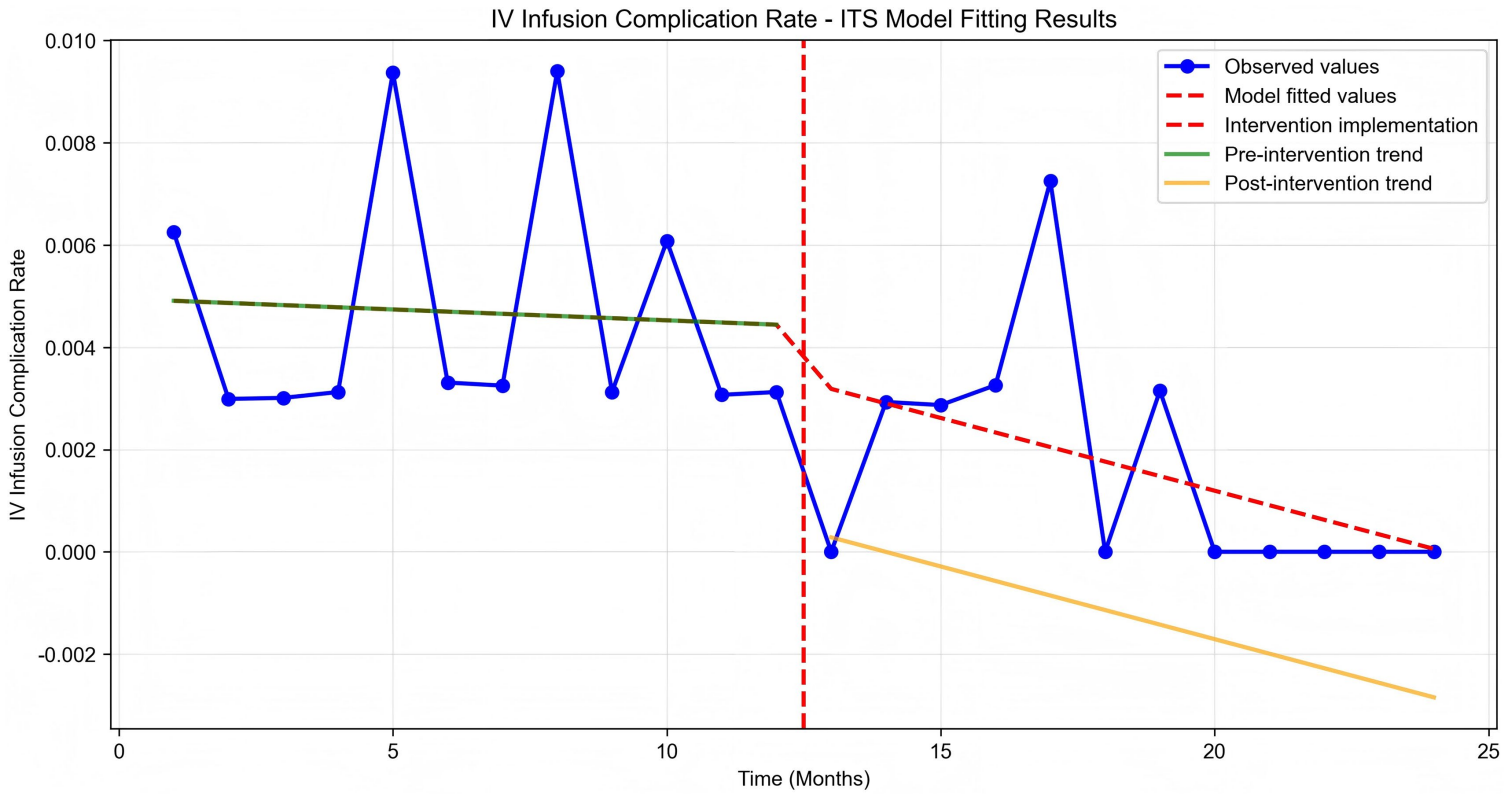
IV infusion complication rate ITS model fitting.

Time series plots were constructed for the incidence rate of intravenous infusion complications over 24 months (Figure 7). Results of the interrupted time series model fitted with Newey-West correction for autocorrelation (Table 3; Figure 8) showed that: Before the intervention, there was no significant change in the monthly incidence rate of infusion complications in the two wards (β1 = 0.00, SE = 0.01, *p* = 0.6704). At the intervention node, there was no significant level decrease in the incidence rate of infusion complications in the two wards (β2 = −0.10, SE = 0.10, *p* = 0.3177). After the intervention, the trend of complication rate showed a slight decrease compared with that before the intervention, and the monthly decrease rate increased by 0.02 percentage points compared with the pre-intervention period. However, this change did not reach statistical significance (β3 = −0.02, SE = 0.01, *p* = 0.2263), indicating that there was still no significant trend of change in the complication rate after the intervention (β1 + β3 = −0.02 percentage points per month, *p* = 0.2263).

In addition, analysis of the relationship between drug ratio and complication rate showed: Model specification: A two-way fixed-effects panel regression model (controlling for ward and month fixed effects) is employed, expressed as: Incidence ijt = *α* + βx Proportion of drug combinations ijt + βxx Monthly hospitalization count ijt + μi + λt + *ε* ijt (where i denotes ward, j drug category, t month; μi represents ward fixed effects; λt month fixed effects; ε ijt denotes the random error term). Baseline regression: The proportion of core drugs for cerebrovascular disease treatment was significantly negatively correlated with the complication rate (coefficient = −0.032, *t* = −3.16, *p* < 0.01), indicating that a higher proportion of core drugs was associated with a lower complication rate. Neither the proportion of infection control and symptomatic treatment drugs (coefficient = 0.007, *t* = 1.70, *p* > 0.05) nor the proportion of intracranial pressure control/dehydration drugs (coefficient = −0.001, *t* = −0.16, *p* > 0.05) had a significant impact on the complication rate (Table 8). Subgroup by ward: After subgroup regression by ward, the correlations between the proportion of core cerebrovascular disease drugs, the proportion of infection control and symptomatic treatment drugs, the proportion of intracranial pressure control/dehydration drugs, and the complication rate were no longer significant in either Ward A or Ward B (all *p* > 0.05). This suggests that the relationship between drug ratio and complications may vary with ward characteristics, and the drug ratio does not significantly affect the complication rate in the two specific wards (Table 9). The sample size for each ward subgroup was 24 observations, with consistent control for month fixed effects and other covariates across analyses.

**Table 8:** Baseline regression analysis.

Variables	(1)	(2)	(3)
	Complication_rate	Complication_rate	Complication_rate
Neuro_drug_ratio	−0.032*** (−3.16)		
Log_pop	0.073 (0.09)	−0.555 (−0.68)	−0.930 (−1.19)
Infection_drug_ratio		0.007 (1.70)	
Icp_drug_ratio			−0.001 (−0.16)
Constant	1.416 (0.36)	2.925 (0.71)	5.059 (1.28)
Observations	48	48	48
R-squared	0.821	0.770	0.754
Firm FE	Yes	Yes	Yes
Year FE	Yes	Yes	Yes
Controls	Yes	Yes	Yes

Robust *t*-statistics in parentheses. ***p < 0.01.

**Table 9:** Regression analysis by ward.

Variables	(1)	(2)	(3)	(4)	(5)	(6)
	A	A	A	B	B	B
	Complication_rate	Complication_rate	Complication_rate	Complication_rate	Complication_rate	Complication_rate
Neuro_drug_ratio	−0.021 (−1.59)					
Infection_drug_ratio		0.052 (1.42)			−0.000 (−0.08)	
Icp_drug_ratio			0.033 (1.64)			0.000 (0.08)
Log_pop	−2.475 (−1.18)	−2.931 (−1.47)	−2.310 (−1.09)	−0.035 (−0.18)	−0.042 (−0.19)	−0.042 (−0.19)
Constant	15.135 (1.54)	15.375 (1.50)	12.176 (1.12)	0.207 (0.20)	0.246 (0.21)	0.232 (0.21)
Observations	24	24	24	24	24	24
R-squared	0.373	0.359	0.377	0.001	0.002	0.002
Ward ID	1	1	1	2	2	2
Month FE	Yes	Yes	Yes	Yes	Yes	Yes
Controls	Yes	Yes	Yes	Yes	Yes	Yes

### 3.4 Patient satisfaction

Independent sample *t*-test was used for comparison of satisfaction scores between groups. A comparison of inpatient satisfaction in the unit before and after system application showed a statistically significant difference in satisfaction scores (*p* = 0.046) (Table 10).

**Table 10:** Patient satisfaction (scores).

Items	Before application (3,126 people)	After application (3,220 people)	t	*p*	SMD
Satisfaction	99.18 ± 5.99	99.45 ± 4.46	−1.995	0.046	−0.05

### 3.5 Abnormal infusion alarm frequency and average response time

Descriptive statistics were used to calculate the number of alarms, alarm composition, and average response time. Total abnormal alerts: 114,793 (air alarms: 4,411, 3.84%; blockage/slow/fast alarms: 85,322, 74.33%; completion alarms: 25,060, 21.83%). The average response time for nurses was 1.81 min for the air alarm, 2.12 min for the infusion blockage/slow/fast alarm, and 2.51 min for the completion alarm. The alarms per device per day was: 114,793 alarms ÷ (99 devices × 365 days) ≈ 3.18 alarms per device per day. The alarm rate per 1,000 infusions was: (114,793 alarms ÷ 55,824 infusions) × 1,000 ≈ 205.6 alarms per 1,000 infusions.

### 3.6 Nurse satisfaction

Descriptive statistics were used to calculate the satisfaction rate. A total of 44 nurses from 2 units were surveyed, out of which 33 nurses expressed satisfaction with the infusion monitoring system, with a satisfaction rate of 75%.

## 4 Discussion

### 4.1 Application of an intelligent intravenous infusion monitoring system can effectively improve the timely completion rate of intravenous infusion

Intravenous infusion accounts for a significant proportion of nursing workload in China, and infusion quality is a core indicator for evaluating nursing safety, effectiveness, and service level (4, 16). The “Healthy China 2030” Planning Outline (2016) and the “National Hospital Informationization Construction Standards and Specifications” (2023) have successively emphasized the strategic importance of improving nursing efficiency through informatization and optimizing service quality (17, 18). In response to these national policy requirements, this study developed an intelligent intravenous infusion monitoring system based on IoT technology, aiming to address the challenges of infusion process management and enhance infusion quality.

Comparative analysis showed that the timely completion rate of intravenous infusion significantly increased after system application: Ward A (Neurology) rose from 28.39 to 38.58%, Ward B (Orthopedics) from 41.52 to 59.52%, and the overall rate from 35.59 to 47.23% (all *p* < 0.001). This confirms that the intelligent monitoring system can effectively improve the timeliness of infusion completion, consistent with the findings of previous studies (10). Further sensitivity analysis results showed that even if it is assumed that all pre-intervention missing data are attributed to non-on-time completion, the system can still significantly improve the on-time completion rate. This further confirms that its effectiveness is not seriously affected by the distribution of missing data, and the results are highly credible.

Further interrupted time series analysis (with Newey-West correction for autocorrelation) revealed deeper temporal trends: before the intervention, the monthly timely completion rate of the two wards showed a significant downward trend (β1 = −0.0189, *p* < 0.001), reflecting the limitations of traditional manual monitoring in maintaining stable infusion quality. After system implementation, the monthly rate increased by 4.56%, with a significant slope elevation of 0.0645 compared to the pre-intervention period (β3 = 0.0645, *p* < 0.001), indicating that the system not only reversed the declining trend but also achieved a sustained and stable improvement in infusion timeliness. Notably, there was no significant level jump at the intervention node (β2 = −0.0563, *p* = 0.259), suggesting that the system’s effect is primarily achieved through continuous process optimization rather than short-term intervention, which is more conducive to long-term nursing quality improvement.

Subgroup analysis further revealed the heterogeneous effects of the system across different wards and drug types. The negative correlation between on-time completion and complication rate in the two wards may stem from differences in ward characteristics: Ward A (Neurology Department) admits more critically ill patients with complex conditions who require strict control of infusion timeliness, so on-time completion can effectively reduce complications. In contrast, Ward B (Orthopedics Department) mainly treats surgical patients with relatively stable conditions, where other factors (such as postoperative rehabilitation status and drug compatibility) have a more significant impact on complications, leading to a positive correlation contrary to the baseline trend. Regarding drug types, only Class D drugs (Edaravone Dexborneol Concentrated Solution for Injection) showed a significant correlation between on-time completion and complication rate, which may be related to its special properties (e.g., this drug requires strict control of infusion rate, and timely infusion is crucial for efficacy and safety). Other drugs did not show a significant correlation, possibly because they have higher tolerance to deviations in infusion time, or clinical dosage and administration routes have been optimized to reduce the impact of time deviations on complications.

The timeliness of infusion directly affects the stability of blood drug concentration, therapeutic efficacy, and the risk of adverse reactions (19). It also reflects the standardization of medical order execution and the overall level of nursing management. The intelligent infusion monitoring system realizes real-time tracking of infusion progress and abnormal alarms (e.g., blockage, speed deviation), enabling nurses to promptly identify and address potential issues. Additionally, the system’s alarm priority classification helps nurses optimize their workflow arrangement, improving work efficiency while ensuring infusion safety. These functional advantages collectively contribute to the significant improvement in the timely completion rate, providing a feasible technical solution for advancing nursing informatization and enhancing the quality of infusion services.

### 4.2 Application of the intelligent intravenous infusion monitoring system can reduce the occurrence of intravenous infusion-related complications

Intravenous infusion-related complications are closely related to the infusion drip rate (20). A rapid drip rate of intravenous fluid infusion may cause excessive cardiac burden, leading to pulmonary edema, or it may increase the risk of catheter blockage, leakage, and infection. A slow drip rate may prevent reaching the desired blood medication concentration, reducing treatment effectiveness (21, 22). The present study utilized the intelligent intravenous infusion monitoring system and revealed that despite a significant increase in infusion volume in both departments after the system was implemented, the occurrence of infusion-related complications in the Neurology department decreased from 17 cases per year to 6 cases per year (*p* = 0.003), while in the Orthopedics department from 1 case per year to 0 cases per year (*p* = 0.279), and overall (Neurology and Orthopedics) from 18 cases per year to 6 cases per year (*p* = 0.002). This indicates that using an intelligent intravenous infusion monitoring system can effectively reduce the occurrence of intravenous infusion-related complications (23).

The analysis of drug ratio and incidence of infusion-related complications further supplemented the understanding of risk factors: the proportion of core drugs for cerebrovascular disease treatment was significantly negatively correlated with the incidence of infusion-related complications. This may be because these core drugs target the key pathological mechanisms of cerebrovascular diseases, and an adequate dosage proportion can better control the primary disease, thereby reducing secondary infusion-related complications. However, the proportion of intracranial pressure control/dehydrating drugs and infection control & symptomatic treatment drugs had no significant effect. This is possibly due to the fact that their dosage has been strictly controlled according to clinical guidelines, so changes in their proportion have little impact on the overall incidence of infusion-related complications. The loss of significance in the subgroup analysis by ward suggests that the effect of drug ratio is regulated by ward-specific factors, such as patient population structure, disease severity, and combined medication strategies. These factors need to be considered in clinical practice.

This system collects and monitors patient infusion data (drip rate, status, volume infused) through a drip rate infusion controller and sends these data in real time to the infusion client, which is then transmitted to a large infusion screen. Additionally, wearable wristwatches worn by nursing staff enable timely notification of abnormal infusion drip rates for patients under their care. Therefore, regardless of their location, nursing staff can be informed of a patient’s infusion status in a timely manner and adjust promptly, standardizing the infusion drip rate and achieving real-time control. The average processing time for rapid/slow drip rate alarms in this study was 2.12 min, effectively reducing the occurrence of infusion-related complications. Similar to the researches by Li and Xiao (6, 24), by monitoring the infusion process and visualizing it, the time taken by nurses to handle abnormal infusion events was shortened, effectively reducing the occurrence of adverse events, including backflow, swelling, leakage, and blockage.

### 4.3 The impact of the intelligent intravenous infusion monitoring system on patient and nurse satisfaction

This study also analyzed the satisfaction of inpatient and nursing staff with the infusion monitoring system. The results showed an overall improvement in patient satisfaction (99.18 versus 99.45, *p* = 0.046). This indicates that the system has a certain effect on improving inpatient satisfaction, similar to the results of a study by Wang (25). This may be because, in traditional infusion processes, patients/families must actively monitor infusion status and notify nurses of abnormalities via calls or bells. The system relieves patients/families from real-time infusion monitoring, as abnormal events are detected and addressed promptly via alerts. This relieves the pressure on patients and their families, who no longer need to monitor the infusion process in real time, thereby increasing patient and family satisfaction.

Notably, despite the statistically significant difference in patient satisfaction scores between the two groups, the standardized mean difference (SMD) suggests a clinically trivial actual difference in satisfaction levels. This discrepancy may be explained by the study’s limitations: as a retrospective study, the patient satisfaction questionnaire adopted in this research evaluates the overall nursing services during hospitalization, with only a small proportion of items specifically targeting infusion-related care. The insufficient specificity of the questionnaire for infusion services may have weakened the ability to capture the true difference in infusion-related satisfaction before and after the system’s application, ultimately resulting in a modest improvement in overall satisfaction.

This study found that nurse satisfaction with the infusion monitoring system was only 75%. While nurses acknowledged improvements in efficiency, quality, and data management, they noted room for optimization in system convenience, intelligence, and sensitivity. This may be due to the lack of full intelligence of the system, the complex and time-consuming process of replacing infusion fluids, and frequent infusion equipment failures. Another reason may be that before the introduction of this system, there were no infusion warnings. Consequently, nurses often overlooked the timing and drip rate compliance of infusions. However, after the introduction of intelligent infusion equipment, nurses had to adjust based on system warning information, which consumed time and led to lower nurse satisfaction than expected.

### 4.4 Cost–Benefit analysis

This section presents an illustrative cost–benefit analysis based on retrospective data and several key assumptions. The calculations are intended for explanatory purposes.

Assumptions: Equipment cost: The annual operational cost for all equipment is assumed to be ¥49,500. Nurse wage: The analysis uses an assumed fully burdened wage of ¥50 per hour for nursing staff. Time conversion: Saved monitoring time is directly converted into cash savings based on the assumed wage rate. Time savings utilization: The calculated time savings (57.75 h/day) are assumed to be fully utilized for other value-adding activities or represent a real reduction in labor requirements. Complication costs: The cost per complication case is estimated at ¥5,000.

Scenario analysis: Given the inherent uncertainties in the underlying assumptions, the following scenarios are presented to provide a more comprehensive perspective (Table 11).

**Table 11:** Cost–Benefit analysis (illustrative).

Scenario	Assumption adherence	Annual direct benefit (time savings)	Annual indirect benefit (complications)	Total annual benefits	Cost–Benefit ratio
Best-case	High time utilization, higher wage (e.g., ¥60/h)	¥1.25 million	¥72 k (further reduction)	¥1.32 million	01:26.7
Base-case (illustrative)	As per initial assumptions	¥1.04 million	¥60 k	¥1.10 million	01:22.2
Worst-case	Lower time utilization, lower wage (e.g., ¥40/h)	¥0.83 million	¥48 k (smaller reduction)	¥0.88 million	01:17.8

Base-case (illustrative) calculation: (1) Annual operational cost: ¥49,500; (2) Annual benefits:

Direct benefits: Manual monitoring time saved is approximately 57.75 h per day (≈1,732.5 h/month), which, under the base-case assumptions, is equivalent to ¥1.04 million per year.

Indirect benefits: A reduction in complications from 18 to 6 cases per year, saving an estimated ¥60,000 per year. (3) Cost–Benefit ratio: Total benefits (¥1.10 million) versus cost (¥49,500) yield an illustrative ratio of 22.2:1.

This illustrative analysis suggests the potential for a significant positive return on investment. However, the actual financial impact is highly sensitive to the key assumptions listed above, particularly the monetization of saved staff time. The wide range of outcomes in the scenario analysis underscores the need for prospective, real-world data collection to validate these preliminary findings.

### 4.5 Strategies for mitigating alarm fatigue

Threshold adjustment: Extend the manuscript’s existing deviation range framework (±15% for routine drugs, ±10% for high-risk drugs, ±5% for volumes ≤50 mL) to alarm thresholds, reducing false alarms in low-risk scenarios. Debounce logic: Build on the system’s real-time monitoring function by adding a 5–8 s window. If transient abnormalities (e.g., drip rate fluctuations caused by patient movement) disappear within this window, no audio/visual alarm is triggered-only an event log is recorded. Transient peak suppression: Leverage the system’s automatic infusion rate adjustment function to ignore drip rate peaks lasting <2 s; alarms are only triggered if abnormal rates persist beyond 2 s.

### 4.6 Prospective audit plan for false positive alarms

Scope and timeline: September 2023–February 2024 (6 months), covering 99 devices and 44 nurses in Ward A (Neurology) and Ward B (Orthopedics). Methods: Extract alarm logs from the infusion monitoring system, synchronize data from the HIS and HaiTai systems, and have two nurses independently judge false positives (alarms triggered without actual infusion abnormalities). Discrepancies are resolved by the hospital’s Intravenous Therapy Nursing Team. Core indicators: False positive alarm rate (number of false positive alarms ÷ total alarms × 100%) and false positive rate by alarm type. Adjustment mechanism: If the false positive rate of a specific alarm type exceeds 30% for two consecutive months, optimize thresholds (e.g., adjust high-risk drug thresholds to ±8%) or debounce windows, followed by a 3-month re-audit to evaluate effectiveness.

### 4.7 Integration with existing literature and practical challenges

This study’s findings are consistent with the development trend of digital health in global clinical practice. For example, Tomobi et al. (19) pointed out that intelligent infusion monitoring technology can significantly improve the safety of infusion therapy in resource-constrained environments, which is consistent with our conclusion that the system reduces complications and improves on-time completion rates. Additionally, the low nurse satisfaction rate observed in this study is also reflected in other similar studies (2), which may be related to the learning curve of new technologies and the increase in short-term workload.

In terms of practical application challenges, the integration of the intelligent infusion monitoring system with existing hospital information systems (HIS) requires technical compatibility adjustments, which may increase the initial deployment cost. Moreover, the maintenance of equipment (such as daily inspection and monthly calibration) requires additional human resources input. For nurses, the transition from traditional manual monitoring to intelligent alert-based work mode requires adaptive training to avoid alarm fatigue. These challenges need to be addressed through multi-departmental collaboration, optimized training programs, and continuous technical upgrades in future promotion.

## 5 Limitations

First, as a retrospective study, it has inherent limitations in causal inference. Despite adjusting for potential confounding factors through statistical methods, it is still impossible to completely rule out the impact of unmeasured confounders (such as patient age, comorbidities, and length of hospital stay). Due to the lack of information storage capability in the previously used hospital-wide call system, relevant data could not be collected to evaluate the impact of the system before and after its application on the frequency of patient call bell usage, which is one of the limitations of this study. In this study, there was a high number of abnormal infusion alerts, with infusion blockage/slow/fast alerts accounting for the highest proportion. High abnormal alert volume, with some potentially caused by system failures, indicating needs for improved intelligence/sensitivity. Notably, although nurses were unaware of the specific research purpose, the deployment of the new alarm device was perceptible to the staff. This may potentially alter their clinical behaviors, such as increasing vigilance in infusion monitoring, indicating that the risk of the Hawthorne effect has been mitigated rather than completely eliminated. Additionally, statistics on the alarm rate, false positive rate, and failure rate of each device were not conducted, which constitutes another limitation of this study. This missing data hinders a comprehensive assessment of the system’s operational performance and the accuracy of alerts, making it difficult to quantitatively analyze the extent to which system failures contribute to abnormal alerts. Future studies could include statistics on relevant data.

## 6 Conclusion

The application of an intelligent intravenous infusion monitoring system can effectively improve the timely completion rate of intravenous infusion and reduce the occurrence of intravenous infusion-related complications. Through real-time monitoring and alarm functions, the system helps nursing staff to promptly detect and address infusion irregularities, improve the management quality of the infusion process for patients, ensure patient infusion safety, and enhance patient satisfaction. The system is applicable to all infusion-requiring patients in medical institutions, especially in precision-dependent departments (pediatrics, oncology, ICU). Although the timely completion rate of intravenous infusion for patients has significantly improved, there remains a potential for improving the intelligence and convenience of the system. Therefore, future research and investment should focus on intelligent infusion equipment to better ensure the safety of clinical patient infusions and enhance nurse work efficiency.

## Data Availability

The original contributions presented in the study are included in the article/supplementary material, further inquiries can be directed to the corresponding author.
